# Applications of Supercritical Anti-Solvent Process in Preparation of Solid Multicomponent Systems

**DOI:** 10.3390/pharmaceutics13040475

**Published:** 2021-04-01

**Authors:** Guijin Liu, Junjian Li, Shiming Deng

**Affiliations:** School of Life and Pharmaceutical Sciences, Hainan University, Haikou 570228, China; dsm701@126.com

**Keywords:** multicomponent systems, supercritical anti-solvent, co-crystals, solid dispersions, solid-state properties, continuous manufacturing

## Abstract

Solid multicomponent systems (SMS) are gaining an increasingly important role in the pharmaceutical industry, to improve the physicochemical properties of active pharmaceutical ingredients (APIs). In recent years, various processes have been employed for SMS manufacturing. Control of the particle solid-state properties, such as size, morphology, and crystal form is required to optimize the SMS formulation. By utilizing the unique and tunable properties of supercritical fluids, supercritical anti-solvent (SAS) process holds great promise for the manipulation of the solid-state properties of APIs. The SAS techniques have been developed from batch to continuous mode. Their applications in SMS preparation are summarized in this review. Many pharmaceutical co-crystals and solid dispersions have been successfully produced via the SAS process, where the solid-state properties of APIs can be well designed by controlling the operating parameters. The underlying mechanisms on the manipulation of solid-state properties are discussed, with the help of on-line monitoring and computational techniques. With continuous researching, SAS process will give a large contribution to the scalable and continuous manufacturing of desired SMS in the near future.

## 1. Introduction

Active pharmaceutical ingredients (APIs) are usually formulated into designed dosage forms before being administered to patients. The performance of dosage forms is strongly influenced by the physicochemical properties of APIs, such as chemical stability, mechanical properties, hygroscopicity, solubility, and dissolution rate [1,2]. Unfortunately, there are many drug candidates with poorly physicochemical properties under development. Thus, it is essential to improve insufficiently physicochemical properties for these drug candidates capable of accessing the market.

The vast majority of drug products are supplied in solid dosage forms, such as tablets, capsules, powders, and dry syrups. The solid-state properties of APIs play an important role in modifying their physicochemical properties [2,3]. It is paramount for the pharmaceutical industries to control accurately the solid-state properties of APIs during their manufacture, formulation and shelf life. In recent years, solid multicomponent systems (SMS) have been of great interest to the pharmaceutical scientists. The SMS development mainly focuses on the manipulation of the solid-state properties of APIs, such as the formation of salts, amorphous formulations, solvates, cocrystals, liquid crystals, and nano-cocrystals, to overcome problems of the final drug form, such as poor solubility and dissolution rate, hygroscopicity, poor tabletability, instability, and bitter taste [4,5].

There are two major existing approaches for the production of solid particles, i.e., top-down approaches and bottom-up approaches [6]. Top-down approaches are essentially high energy processes where drug particles are generated through the comminution of bulk materials by the use of technologies such as jet milling, pearl-ball milling, and high-pressure homogenization [7]. On the contrary, the bottom-up approaches are broadly called precipitation processes where drug particles are created from molecules or nucleus by the use of technologies such as spray drying, anti-solvent precipitation, and hot melt extrusion [8]. In general, top-down approaches present several challenges, including a limited control over the solid-state properties of APIs, a long processing time to achieve the required size and the thermal/mechanical degradation of temperature-sensitive APIs. Bottom-up approaches through controlled crystallization or precipitation represent an emerging area in manipulating the solid-state properties of APIs. Among them, supercritical fluid (SCF) techniques have been investigated and applied by many researchers and companies for the solid-state pharmaceutical development [9,10,11,12]. SCFs exhibit properties in between a vapor and a liquid. That is, their density is similar to a liquid, allowing for a good solvation power, while viscosity and diffusivity are similar to that of a vapor, allowing for efficient mass transfer. SCFs, in particular supercritical carbon dioxide (scCO_2_), provide numerous opportunities for the development of ideal particle formation processes in the pharmaceutical industry. ScCO_2_ is safe, inexpensive, readily available, and an ideal substitute for many toxic solvents. ScCO_2_ was first applied to extraction of natural products at the end of the 1980s. Nowadays, scCO_2_ based extraction technology has become a mature and readily controlled process in the extraction industry, which has been utilized as a green approach for the productive extraction and recovery of valuable compounds, such as bioactive plant phytochemicals [13], nutrition and functional food ingredients [14], and other high added-value compounds [15]. However, the development of the scCO_2_ technology for the production of solid particles in the pharmaceutical industry is still in the early stages. ScCO_2_ can act as solvent, anti-solvent, or solute at different processes. Well-established scCO_2_ processes include rapid expansion of supercritical solution (RESS) process, particles from the gas-saturated solution (PGSS) process, and supercritical anti-solvent (SAS) process [16,17,18].

SAS process, by exploiting the anti-solvent effect of scCO_2_, is a potential alternative to conventional anti-solvent crystallization, which stands out for the production of solid particles of one or more compounds in a controlled manner [19,20,21,22]. By utilizing the tunable properties of scCO_2_ at different operating conditions, SAS process offers the possibility to control the crystalline form of APIs in the micron, sub-micron and nano ranges, then to produce nanoparticles, microparticles, expanded hollow microparticles, micro/nano crystals, amorphous particles, and others [23,24,25,26]. In our previous studies, SAS process has been applied to produce 10-hydroxycamptothecin proliposomes [27], 10-hydroxycamptothecin/poly (L-lactic acid) microspheres [28], camptothecin microcrystals [29], gefitinib polymorphs [30], paracetamol/trimethylglycine co-crystals [31], nimesulide amorphous solid dispersions [32], and itraconazole solid dispersions [33]. These studies have demonstrated that SAS process holds great promise for the manipulation of the solid-state properties of APIs.

**Figure 1:**
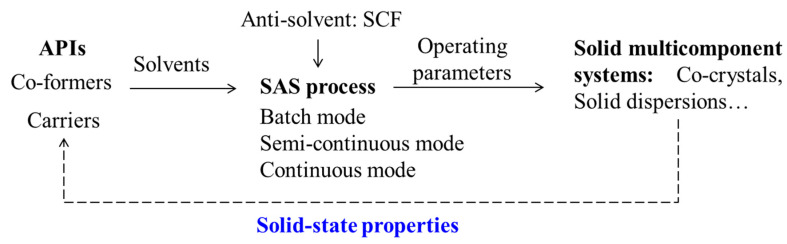
An overview of the SAS applications for solid multicomponent systems.

Combining with research experiences in our research group, this paper aims to provide the reader with a comprehensive review of the applications of SAS process in the SMS preparation. An overview of the process is shown in Figure 1. The development of SAS process from batch to continuous is introduced firstly. Then, examples of pharmaceutical co-crystals and solid dispersions prepared via the SAS process are summarized. After that, the underlying mechanisms on the manipulation of solid-state properties of APIs are discussed. Finally, guidelines for future prospects are proposed.

## 2. SAS Process: From Batch to Continuous

In the SAS process, SCF acts as an anti-solvent and causes the precipitation of APIs and/or excipients from organic solvents. Nowadays, the SAS technique has been developed in batch, in semi-continuous, and more recently in continuous mode.

### 2.1. Batch Mode

**Figure 2:**
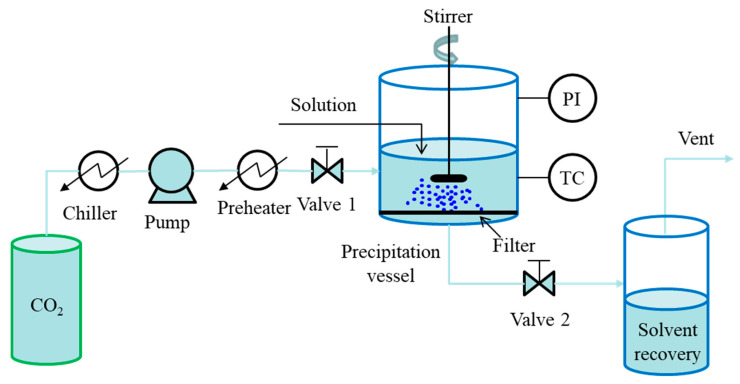
Schematic diagram of batch SAS process: GAS process.

The batch mode is known as the gas anti-solvent (GAS) process, which has been proposed in 1989 by Gallagher et al. [34]. A simplified diagram of the GAS process is presented in Figure 2. In general, the liquid solution is prepared and poured into the precipitation vessel firstly. Then, compressed CO_2_ is pumped at a given flow rate into the vessel through valve 1 for pressurization, where valve 2 remains closed, and pressure inside the vessel is measured, and temperature is controlled. During the pressurization, the solvating power of solvent is reduced, resulting in the precipitation of the dissolved solute. After the formation of desired particles, valve 2 is opened for solvent removal and CO_2_ flushing. Finally, the obtained particles are collected from the filter located at the bottom of the vessel for further analyses. In particular, a stirrer is necessary to improve the mixing between the solution and the scCO_2_ when relatively large volumes of solution are processed. Additionally, a sapphire window on the base of the vessel with the fiber optic lighting is useful for visual observation of the process [35]. The GAS process is simple and particularly useful for the crystallization of pharmaceuticals. However, a clear disadvantage of the GAS process is its batch mode, resulting in a relatively small production capacity, which is limited by the capacity of the precipitation vessel.

### 2.2. Semi-Continuous Mode

**Figure 3:**
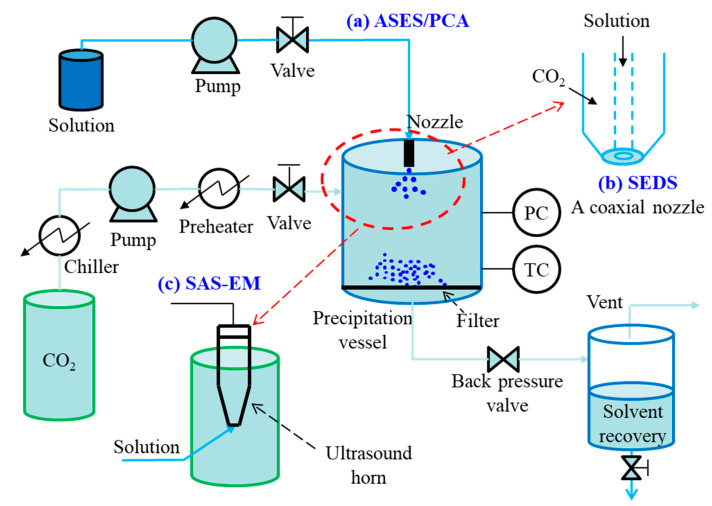
Schematic diagram of semi-continuous SAS process: (**a**) ASES/PCA, (**b**) SEDS process, (**c**) SAS-EM process.

The semi-continuous mode is known as aerosol spray extraction system (ASES) [36] or precipitation with compressed anti-solvents (PCA) [37]. A simplified diagram of the semi-continuous SAS process is presented in Figure 3. In general, liquefied CO_2_ is continuously introduced into the precipitation vessel via a high-pressure pump at a given flow rate, meanwhile pressure and temperature inside the vessel are controlled. When the desired temperature and pressure are reached, pure solvent is sprayed into the precipitation vessel through a nozzle for few minutes to obtain steady state composition conditions of the fluid phase during the solute precipitation. Then, the solution is injected instead of pure solvent into the precipitation vessel at a given flow rate. When in contact with the scCO_2_, the liquid solution including solute dissolves into the scCO_2_, so high supersaturation of solute in the mixed solution can be achieved, resulting in the precipitation of the dissolved solute. At the end of the solution delivery, scCO_2_ is kept flowing to remove the residual solvent. Finally, the obtained particles are collected from the precipitation vessel after the pressure relief.

In ASES or PCA, to minimize particle agglomeration frequently observed and to reduce or eliminate drying times, increased mass transfer rates are required. Some modified versions are proposed, such as solution enhanced dispersion by supercritical fluids (SEDS) process [38], and supercritical anti-solvent with enhanced mass transfer (SAS-EM) process [39]. The main difference among these processes is in the injection device. With respect to ASES/PCA, SEDS process consists of a two (or three) coaxial passages nozzle to provide a simultaneous introduction of the solution/suspension and different solvents; whereas, the SAS-EM process utilizes an ultrasound horn to provide a different methodology to create the jet break-up. The main advantage of semi-continuous SAS techniques over GAS technique is their continuous injection of solution and scCO_2_, which is a prerequisite for large scale mass production of particles. They are also better for the control of particle solid-state properties, such as size, morphology, crystal habits, and others.

### 2.3. Continuous Mode

The continuous mode is known as atomization and anti-solvent (AAS) process [40] or atomization of supercritical anti-solvent induced suspensions (ASAIS) process [41]. The AAS process is similar to SEDS process: for SEDS setup, the solution is atomized into the precipitation vessel pressurized with scCO_2_; but for AAS process, the solution is mixed with the scCO_2_ inside the coaxial nozzle to precipitate solids, and then the solids are collected in the precipitation vessel at near atmospheric pressure.

**Figure 4:**
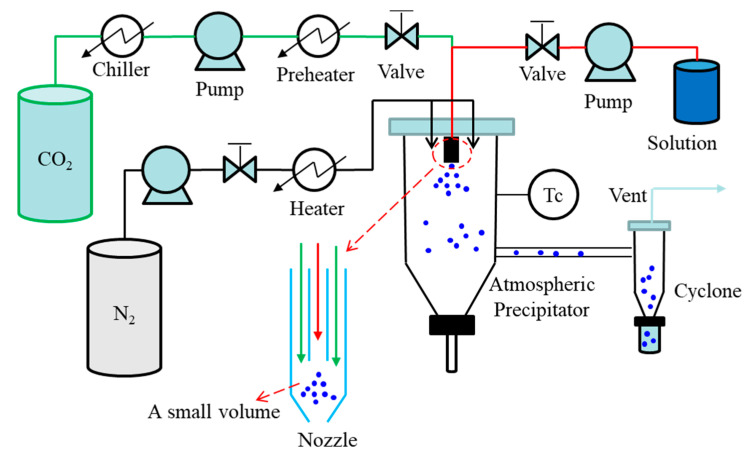
Schematic diagram of continuous SAS process: ASAIS process.

In the pharmaceutical industry, spray drying is a continuous unit operation capable of transforming solutions or suspensions into a solid product. The spray drying process consists of four basic stages: atomization of the liquid by a kinetic energy or pneumatic nozzle where the liquid stream is broken in small droplets by interaction with a second fluid, usually pressurized air; mixing of the droplets with the drying gas, often air or in some cases nitrogen; evaporative drying of the droplets into fine particles; and separation of the dried particles from the gas using a cyclone or a bag-filter [42,43]. The ASAIS technique is a combination of AAS and spray drying. A simple diagram of ASAIS process is presented in Figure 4. In ASAIS, the solution is mixed with scCO_2_ in a small volume mixer inside a coaxial nozzle to generate a suspension which is then immediately sprayed for solvent extraction by spray drying at normal pressure.

The transition of SAS process from batch to continuous is encouraged by most regulatory bodies for pharmaceutical manufacturing processes. In AAS and ASAIS methods, the supercritical conditions are restricted to a very small volume mixer, avoiding the large volume equipment at high pressure and complex particle harvesting in filters. These are compatible with the continuous regime operation and implement at the industrial scale. Moreover, their installations are simplified and become compatible with existing spray drying equipment. When compared to conventional spray drying methods, the continuous SAS techniques present their own irreplaceable advantages. For example, conventional spray drying methods typically generate amorphous APIs, while the continuous SAS techniques have the potential to generate and control the crystalline form of APIs by inducing nucleation inside the nozzle before the spray drying step [11].

## 3. Applications of SAS Process for Solid Multicomponent Systems

### 3.1. Pharmaceutical Co-Crystals

Pharmaceutical co-crystals are defined by the United States Food and Drug Administration (FDA) as crystalline materials composed of two or more molecules (APIs and co-formers) in the same crystal lattice [44]. Co-formers are usually molecules that are not pharmaceutically active, but can interact with the APIs by non-ionic or non-covalent bonds. However, in case of multidrug co-crystals, co-formers with therapeutic activity are used. Pharmaceutical co-crystals have provided opportunities for engineering solid-state forms beyond conventional solid-state forms of an API, such as salts and polymorphs. These alternative crystal structures present better physicochemical properties of clinical relevance. Nowadays, co-crystallization has become a powerful technique to improve the physicochemical properties of APIs.

Co-crystallization using scCO_2_ as a co-solvent or anti-solvent can offer advantages over conventional co-crystallization including a greener solvent choice, a milder conditions avoiding API degradation, and the production of small, uniform particles without additional micronization. Recently, the use of scCO_2_ to elaborate co-crystals is gaining extensive attention and has been reported in many published works. Pando et al. [45] have reviewed various supercritical co-crystallization methods, and their advantages and disadvantages in 2016. Recently, MacEachern et al. [22] published a review focused on exploring critical co-crystallization parameters and feasibility of SCF techniques.

Due to its similarities to conventional anti-solvent processes, GAS process is the most widely reported SCF co-crystallization process. It has been applied to prepare pharmaceutical co-crystals like carbamazepine/nicotinamide, itraconozole/succinic acid, itraconozole/L-malic acid, naproxen/nicotinamide, aminosalicylic acid/nicotinamide, sulfamethoxazole/L-malic acid, ketoconazole/4-aminobenzoicacid, mefenamic acid/paracetamol, mefenamic acid/nicotinamide, resveratrol/isoniazid, and resveratrol/nicotinamide [22]. The main factors that can impact the solids obtained by GAS process include the ratio of API/co-former (*R*), solvent choice and solvent volume (*V*), temperature (*T*), pressure (*p*), solute concentration (*C*), scCO_2_ dosing rate (*F_CO2_*), and stirring speed. Many studies have proved that co-crystal production by GAS process can reduce the thermal and mechanical stress applied to the API compared to grinding processes, reduce organic solvent use compared to traditional solution-based methods by replacing the anti-solvent with environmentally benign CO_2_, allow facile recycling of the organic solvent by simple depressurization, and has the potential to produce co-crystal powders lower in residual solvent compared to solvent evaporation.

However, GAS process may produce co-crystals with large and irregular size. In the study of Ober et al. [35], the itraconazole/L-malic acid co-crystals produced by GAS process were larger particles than those precipitated with *n*-heptane. In the work of Neurohr et al. [46], the obtained naproxen/nicotinamide co-crystals were larger than 100 μm, and varying the operating parameters did not help at reducing particle size effectively. Process simulation of Erriguible et al. [47] demonstrated that co-crystal particles were formed mostly through secondary nucleation during the GAS process. In general, the dominance of secondary nucleation is in accordance with low levels of supersaturation, resulting in the form of large particles.

Semi-continuous SAS process is the second most common reported method for co-crystals production in SCF. It has been applied to prepare pharmaceutical co-crystals like indomethacin/saccharin, diflunisal/nicotinamide, paracetamol/dipoclinic acid, naproxen/nicotinamide, carbamazepine/saccharin, paracetamol/trimethylglycine, paracetamol/5-nitroisophthalic acid, and 5-fluorouracil/(urea, thiourea, or pyrazinamide) [22]. The semi-continuous SAS process is more complicated than GAS process, because it involves the hydrodynamics of the injection apart from the anti-solvent mechanism. Thus, besides *R*, solvent, *C*, *T*, *p* and *F_CO2_*, the nozzle diameter (*D*) and solution flow rate (*F_s_*) can impact the solids obtained by the semi-continuous SAS process. As the continuous atomization of solution during injection, the semi-continuous SAS process often obtains co-crystals with small particle size. For example, paracetamol/trimethylglycine co-crystals < 10 μm were obtained by Zhao et al. [31], which were much smaller than those obtained using the ball milling process. In the study of Cuadra et al. [48], carbamazepine/saccharin co-crystals obtained using methanol as a solvent exhibited heterogeneous sizes with widths varying from 5 to 10 μm, while co-crystals obtained using ethanol and dichloromethane were much smaller. However, small particle size is not a guarantee of semi-continuous SAS process. For example, the naproxen/nicotinamide co-crystals prepared by Neurohr et al. [49] exhibited a thin plate-like morphology and a size distribution ranging 20 μm–1 mm, regardless of the operating conditions.

Up to now, there is only one report about the co-crystal production by continuous SAS process. In the study of Padrela et al. [40], pure indomethacin/saccharin co-crystals were obtained by AAS process. The results showed nearly spherical particles were obtained in the AAS technique, but larger size and elongated shape were characteristics of particles produced by the ASES technique. The particle size distribution of these particles was 0.2–5 μm. As mentioned before, AAS crystallization may be governed by the anti-solvent power or spray drying. In the indomethacin/saccharin co-crystal experiments, the spray drying mechanism prevails. Although there is limited literature on co-crystallization by the continuous SAS process, it will be a valuable tool in the production of co-crystals with a small and uniform particle size.

Some typical examples of pharmaceutical co-crystals prepared via the SAS process are summarized in Table 1, which demonstrates the capacity of SAS process in the manipulation of the solid-state properties of APIs by controlling the operating parameters.

In most cases, co-crystals obtained by the SAS process are found to exhibit the same stoichiometry and crystalline phase as the co-crystals obtained by conventional methods. For example, Ober et al. [35] obtained itraconazole/L-malic acid co-crystals via GAS process, which showed a similar crystal structure to co-crystals produced using a traditional liquid anti-solvent, *n*-heptane. In the study of Cuadra et al. [54], co-crystals of the anti-inflammatory drug diflunisal and nicotinamide obtained via SAS process exhibited the same crystal structure, melting point and FT-IR spectrum as those previously obtained by liquid assisted ball mill grinding and solution crystallization. Pessoa et al. [52] prepared resveratrol/isoniazid (RES/INZ) and resveratrol/nicotinamide (RES/NIC) co-crystals by GAS process, where the crystal structure of RES/INZ co-crystal was in accordance with those reported by Zhou et al. [55] using the liquid-assisted grinding technique, and the crystal structure of RES/NIC co-crystal was in accordance with that reported by He et al. [56] using the evaporation of a mixture of organic solvents (acetone, hexane and toluene).

Sometimes, the co-crystal powders obtained by SAS methods may contain an unquantified amount of amorphous material or homo-crystals. For example, the itraconazole/L-malic acid co-crystal powders obtained by Ober et al. [35] were suspected to contain an unquantified amount of amorphous material, due to their decreased X-ray diffraction peak intensities and differing melting points. Non-cocrystallized components were also found in the resveratrol/nicotinamide co-crystals prepared by Pessoa et al. [52]. In the study of Neurohr et al. [49], when the scCO_2_/solution flow ratios increased to 36 (wt basis), a substantial apparition of naproxen crystals arose, leading to heterogeneous powders made of naproxen/nicotinamide co-crystals and naproxen homo-crystals were produced.

Besides that, the capability of SAS co-crystallization methods to produce the desired polymorphic form was assessed in the study of Cuadra et al. [48]. They found that variation of operation parameters could produce different precipitate outcomes. Pure carbamazepine/saccharin co-crystals polymorph I were obtained using methanol, at 40.0 °C; whilst at 60.0 °C, or using ethanol and dichloromethane, mixtures of polymorphs were obtained.

Moreover, the SAS process can be used to produce the co-amorphous system, which is characterized as a completely miscible single phase amorphous solid system composed of binary- or multi-components in solid state. In the study of Park et al. [57], glimepiride/L-arginine co-amorphous formulation was successfully prepared by the SAS process. The obtained co-amorphous formulation present many pharmaceutical advantages such as small particle size, enhanced solubility, increased dissolution rate, and highly hypoglycemic effect of glimepiride. On the contrary, other comparable formulation methods such as physical mixing and solvent evaporation failed to produce pure glimepiride/L-arginine co-amorphous formulation and melt quenching method induced chemical decomposition of glimepiride.

### 3.2. Solid Dispersions

A pharmaceutical solid dispersion is the dispersion of one or more APIs in an inert carrier matrix at solid state, where the APIs can exist in crystalline, semi-crystalline or amorphous state [58]. Solid dispersions have been developed for many purposes such as increasing the dissolution rate of poorly water-soluble drugs, controlling the release of drug in a desired quantity and location, and modifying the surface properties of drug particles. Based on their composition and purpose, the solid dispersions can be classified into four generations [59]: the first generation is crystalline solid dispersions, prepared using crystalline carriers such as urea and sugars; the second generation is the amorphous solid dispersions, containing amorphous carriers which are mostly polymers; the third generation introduces the surface active agents or self-emulsifiers on the basis of the second generation; and the fourth generation is a controlled release solid dispersion, fulfilled by using the water insoluble polymers or swellable polymers.

Solid dispersions can be produced by various processes, such as co-grinding, spray drying, anti-solvent precipitation, and melt extrusion [7,8]. The SAS process has been widely exploited for several decades to develop solid dispersions for various medical applications, and hundreds of related papers have been published [20,60].

For a pharmaceutical solid dispersion, the carrier has a significant impact on its properties, especially the drug release behavior. Various carriers have been applied for the solid dispersions prepared via the SAS process, including polyvinylpyrrolidone (PVP), β-cyclodextrin (β-CD), hydroxypropylmethyl cellulose (HPMC), polyethylene glycol (PEG), polylactic acid (PLA), Eudragit polymers, zein, and others. Hydrophilic polymers are often used to enhance the drug solubility and improve the drug dissolution rate. For examples, in the study of Li et al. [61], the hydrophilic polymer PVP K17 was used as the drug carrier matrix to prepare oridonin solid dispersions via GAS technique, which significantly increased the drug dissolution rate of oridonin. With the enhanced dissolution of oridonin, the oral bioavailability of oridonin solid dispersions was dramatically increased to 26.4-fold that of the mixture of oridonin and PVP K17. In the study of Adeli [62], a hydrophilic non-ionic surfactant Pluronic^®^ F-127 was used to prepare irbesartan (IRB) solid dispersions based on SAS process, which improved IRB solubility more than 13 times and increased the IRB dissolution rate greatly. In reverse, hydrophobic polymers are often used to sustain or prolong the drug release. For examples, in the study of Franco et al. [63], zein was used to prepare diclofenac sodium solid dispersions via SAS process. They found that the higher the polymer/drug ratio, the slower was the release, where the systems zein/diclofenac 30/1 reached 90% of drug release in about 85 h, so prolonging the drug release effectively. In the study of Franco and De Marco [64], Eudragit L100–55 was selected as the polymeric carrier for obtaining diclofenac (DICLO) and theophylline (THEOP) solid dispersions via the SAS process. Dissolution tests showed that the release of the drugs was significantly delayed, up to 28 and 57 times for DICLO and THEOP, respectively.

Some typical examples of solid dispersions prepared via the SAS process are summarized in Table 2. Various solid dispersions with desired solid-state of APIs can be fulfilled by controlling the SAS operating parameters.

Although GAS process has been applied to produce solid dispersions in some references, its application is limited by the poor solid-state properties of obtained particles. In general, the solid dispersions produced by GAS process are irregular, large and agglomerate. In most cases, the obtained solid dispersions possess the crystalline structure of raw APIs or have excess drugs on their surface. For example, in the study of Dittanet et al. [65], the obtained mefenamic acid/PVP K30 composites showed particle agglomeration and growth, appeared to be porous and irregularly shaped, and possessed the crystalline structure of mefenamic acid. In the study of Charoenchaitrakool et al. [66], the obtained theophylline/PEG 4000 composites after GAS process were a mixture of PEG particles and some rectangular shape of theophylline particles, which needed a further washing step with ethyl acetate to remove the excess drugs.

**Figure 5:**
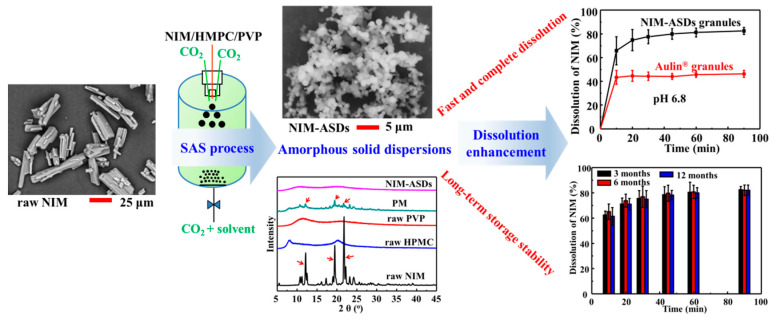
Nimesulide amorphous solid dispersions produced by SAS process. Reproduced with permission from [32], Elsevier, 2020.

With respect to GAS process, the solution is continuous sprayed through a nozzle as fine droplets into the precipitation vessel for the semi-continuous SAS process. This accelerates the mass transfer between scCO_2_ and solution, which is beneficial to produce particles with better solid-state properties. In fact, many published works have demonstrated that the semi-continuous SAS process offers many advantages to produce solid dispersions if compared with conventional techniques [70,71,72]. Depending on carriers and operating conditions, the obtained solid dispersions could be irregular and/or coalescing particles, micro-particles, nanoparticles, crystals, coexistence of crystals or films. In most cases, amorphous particles with spherical shape, small size, and narrow distribution were obtained at the suitable conditions. Fundamentally, the amorphous solid can minimize crystal packing energy by disrupting the drug crystal lattice, which usually achieves the highest level of solubility, and has a faster dissolution rate with respect to the crystalline state [73,74]. For example, in the study of Park et al. [67], the mean particle size of obtained tadalafil/PVP solid dispersions varied from 200 nm to 900 nm, where the drug concentration *C* was the dominant experimental variable on the particle solid-state properties. When *C* = 15 mg/mL, a crystalline form of tadalafil with irregular, micron-sized particles was obtained, and resulted in low dissolution rate. But when *C* = 5–10 mg/mL, an amorphous form of tadalafil with spherical, nano-sized particles was obtained, and resulted in fast and high drug dissolution rate. In the work of Franco and De Marco [64], the semi-continuous SAS process was used to coprecipitate Eudragit L100-55 (EUD) with diclofenac and theophylline, where pherical microparticles with small particle size were obtained at suitable conditions, and the coprecipitated powders were characterized by an amorphous behavior. In one of our previous work [32], we found that coprecipitation with HPMC and PVP varied the nimesulide (NIM) morphology effectively, and NIM could exist in crystal forms or become amorphous in different operating conditions. Under a suitable condition, amorphous powders formed by well separated spherical microparticles were obtained, which increased the NIM solubility more than 5-folds, resulting in faster and more complete NIM dissolution than the commercial Aulin^®^ granules (Figure 5).

Up to now, there are limited reports about the production of solid dispersions by continuous SAS process. Thanks to its similarity and compatibility to spray drying, the continuous SAS process is expected to produce solid dispersions with unique superiority. In fact, the study of Long et al. [75] have demonstrated that the polymorphic outcome of carbamazepine (CBZ) were controllable by using different additives in a continuous SAS process: (1) when using ethyl cellulose as an additive, amorphous CBZ was obtained; (2) when using either maltitol or L-Eudragit 100-55 as additives, pure CBZ form I was obtained; (3) when using sodium stearate as additive, pure CBZ form II was obtained; and (4) when using sodium dodecyl sulfate as an additive, pure CBZ form III was obtained. However, only CBZ form IV was consistently produced by conventional spray drying, irrespective of the additive selected.

## 4. Discussion of the Manipulation of the Solid-State Properties of APIs

From the above examples of co-crystals and solid dispersions prepared via the SAS process, it can be found that the particle solid-state properties are well manipulated by controlling the operating parameters. In this section, the underlying mechanisms on the influence of operating parameters are discussed, where on-line monitoring and computational techniques are useful to rationalize and build the relationship between SAS processing and particle solid-state properties.

### 4.1. Influence Mechanisms of Operating Parameters

The fundamental mechanisms of the formation of co-crystals and solid dispersions via the GAS process are much similar to the conventional anti-solvent crystallization. GAS process consists of the addition of the SCF to a solution, which determines an expansion of the liquid phase. During the volume expansion, the solute separates from the liquid phase as a result of the reduction in solvation power. The relative total volume expansion (∆*V*/*V*) of the liquid phase used in GAS process has been defined by Gallagher et al. [34] as:

$$\frac{∆V}{V}\;=\;\frac{V_{L}\left(T,\;P,x_{1}\right)-V_{2}\left(T,\;P_{0}\right)}{V_{2}\left(T,\;P_{0}\right)}$$

where *V_L_* is the total volume of the liquid phase, *V*_2_ is the total volume of the pure solvent at the same temperature *T* and reference pressure *P*_0_ (normally atmospheric pressure), and *x*_1_ is the molar fraction of anti-solvent in the solution. As a better criterion for selection of the solvent and the optimum process condition for the GAS process, the modified volume expansion was further proposed by de la Fuente Badilla et al. [76] and Mukhopadhyay [77]. Anyway, the volume expansion of a certain solvent is mainly affected by the *p*, *T* and *x*_1_. In general, the volume expansion increases slowly at low *p* and then rapidly increases at high *p*; the isothermal plots of ∆*V*/*V* versus *p* are different for different *T*; and there is an exponential increase in *V_L_* with increasing *x*_1_. During the GAS process, *p* and *x*_1_ can be controlled by adjusting the CO_2_ dosing rate. A faster dosing rate means reaching the final *p* and *x*_1_ faster, resulting in a higher nucleation rate and formation of smaller particles, which agrees with what is observed for conventional crystallization. The understanding of the volume expansion provides the ability to manipulate the solid-state properties of APIs by GAS process. However, a universally robust process for different systems does not exist.

**Figure 6:**
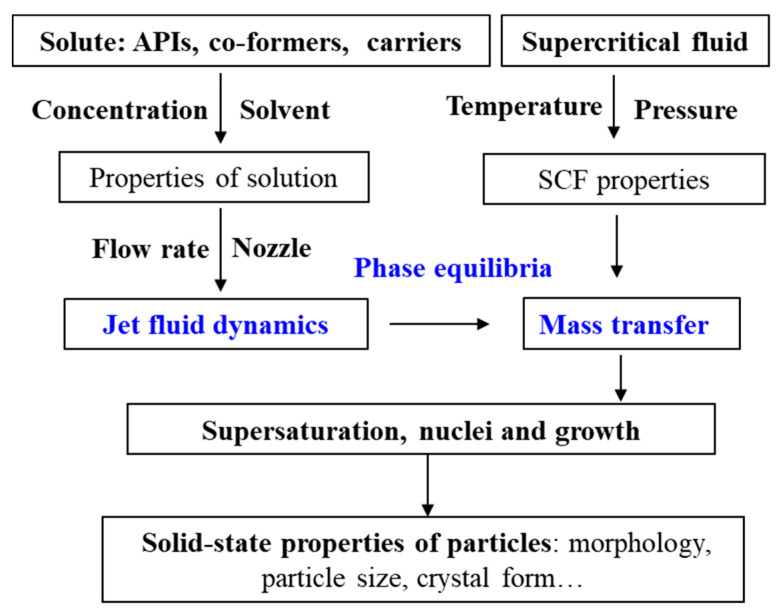
Influence mechanism of operating parameters on the solid-state properties of particles produced by the semi-continuous SAS process.

The semi-continuous SAS process is a complex hybrid technique, which consists of the injection step and anti-solvent precipitation. It mainly involves the high-pressure phase equilibria of the system (solutes, solvents and SCF), fluid dynamics of the injected solution in contact with SCF, mass transfer between the injected solution and SCF [78,79]. The influence mechanism of operating parameters on the solid-state properties of produced particles is illustrated in Figure 6, which involves the mutual interaction of fluid dynamics, phase equilibria and mass transport, and their influence on nucleation and growth mechanisms.

For the phase equilibria, the binary solvent–SCF system is usually used to replace the ternary system (solute–solvent–SCF), because the solubility of solutes in the mixture of solvent and SCF is negligible in most SAS cases. Reverchon et al. [23,24,25,80] correlated three different particle morphologies to the position of the semi-continuous SAS operating point relative to the binary mixture critical point (MCP): nanoparticles can be precipitated at pressures far above the MCP; microparticles can be obtained near above the MCP; whereas expanded microparticles (in some cases called balloons) are produced at sub-critical conditions.

Some authors attempted another explanation of semi-continuous SAS mechanisms starting from the analysis of the fluid dynamic behavior of the solution injected into the pressurized anti-solvent. Dukhin et al. [81] introduced two characteristic times and their competition to describe the appearance of one-phase or multi-phase mixing after jet break-up: a jet break-up time (τ_jb_) and a surface tension vanishing time (τ_i_). If τ_i_ < τ_jb_, near the nozzle orifice a short jet is present, then, gas like jet mixing is obtained in the absence of any interface, where nanoparticle formation by “gas to particle” precipitation is often observed. Instead, if τ_i_ > τ_jb_, a real interface exists between the liquid and the fluid phase, and jet break-up prevails transforming the liquid jet in droplets, where microparticle formation by micrometric droplets drying is the prevailing process. In the study of Marra et al. [82], τ_jb_ was evaluated by solving continuity and conservation of momentum equations; and τ_i_ was evaluated according to the time-evolution model. Based on the time scale approaches, the formation mechanisms of amorphous particles can be well analyzed. However, Rossmann et al. [26] indicated that this time scale model is not applicable for systems forming crystalline structures. They proposed the saturation solubility of the solute in mixtures of solvents and anti-solvents as the indirect classification criterion to distinguish amorphous precipitating or crystallizing.

When droplets are formed, as a result of the jet break-up, mass transport of scCO_2_ into the droplet and solvent evaporation into the bulk scCO_2_ are the two phenomena that characterize the semi-continuous SAS process. Numerical modeling of mass transfer has been established by Werling and Debenedetti [83,84]. In their study, a droplet radius was defined according to the difference in density between the solvent-rich and the antisolvent-rich regions. The droplet radius is a useful parameter for describing the extent of mass transfer and for determining the effect of process conditions on diffusion.

The continuous SAS process is developed from the semi-continuous SAS process and spray drying. Due to the limited references, the influence mechanisms of operating parameters involved in the continuous SAS process are not clear, but can be preliminary analyzed on the basis of those mechanisms involved in the semi-continuous SAS process and spray drying. In the continuous SAS process, nano-suspension is produced in a small volume mixer before the nozzle orifice firstly. This step is similar to the semi-continuous SAS process, and it is possible to control the solid-state properties of APIs by controlling the supersaturation/nucleation events. In general, the ratio of SCF to solution is considerably high in the continuous process when compared to the values obtained in the batch or semi-continuous SAS experiments, strongly ensuring that anti-solvent nucleation are occurring in the nozzle [75]. The generated nano-suspension is then spray-dried after the nozzle orifice. This step is similar to the spray drying, which generates micro- to nano-droplets and also provides the precipitation of existing biocompatible carriers/excipients for APIs encapsulation. An important aspect that distinguishes the continuous SAS process from conventional spray drying concerns the unique precipitation mechanism induced by the anti-solvent effect of SCF in the coaxial nozzle, which provides a unique feature to spray drying processes to control drug polymorphism. Although there are only few reports, the continuous SAS process has shown great potential to produce micro- and nano-particles in one single step and provide accurate control on their final size and solid-state form [11].

### 4.2. Applications of On-Line Monitoring and Computational Techniques

Process analytical technology is a means to produce consistently high-quality products through in-line or on-line analysis, which has been strongly encouraged by the FDA and the ICH [85]. Some on-line monitoring and computational techniques have been well applied in the SAS process for better understanding the process mechanisms, and therefore to be more effective in manipulating the solid-state properties of APIs. Some examples are summarized as follows.

Elastic light scattering was used to study the jet characteristics during SAS precipitation by Reverchon et al. [78]. This technique allows distinguishing between liquid-gas phase boundaries and the formation of a gas-like jet without phase boundaries. They found that light scattering could originate from three different phenomena when a pure solvent was injected into scCO_2_, e.g., jet break-up into rather large droplets, jet atomization into small droplets, and “gas-plume” without droplets. This can be further associated with the mixture critical point and characteristic times discussed above. That is large particles are obtained from large droplets via jet break-up at subcritical conditions; microparticles are obtained from small droplets via jet break-up at near supercritical conditions; and nanoparticles are produced via a gas-to-particle formation process at far above supercritical conditions.

Two-dimensional laser Raman scattering and elastic light scattering was used to analyze mixture driven particle formation in the SAS process by Braeuer et al. [86]. In the SAS process, the particle formation is directly related to the degree of supersaturation, which is a function of the anti-solvent partial density and the mixture composition. This technology qualified for high-resolution, quantitative two-dimensional measurement of the anti-solvent partial density, and of the scCO_2_ mole fraction. Simultaneously, the occurrence of particle formation could be analyzed by the elastic light scattering imaging. This technique was also applied by Dowy et al. [87] to obtain information on the composition distributions and phase boundaries in the highly dynamic SAS mixing process for understanding the influence of different solution concentrations. They indicated that larger particles in the μm-range were produced for higher solution concentrations as a result of the large growth time for the nucleus and the high mass transport rates in liquid-like systems; in contrast, the size of the precipitated particles is smaller at lower solution concentrations, as the shorter time scale for particle growth and lower mass transport rates in gas-like systems.

Foerster resonance energy transfer (FRET) spectroscopy was applied for the in situ measurement of volume expansion of a liquid solution by Braeuer et al. [88]. As discussed above, volume expansion is a key index to manipulate the solid-state properties of APIs via SAS process. Their results indicted the relationship between volume expansion and the ratio of acceptor/donor fluorescence shifts. That was that the decay of acceptor/donor fluorescence ratio was in accordance with the rise of volume expansion, proving the applicability of FRET to measure liquid-phase volume expansion.

An online hyphenation of SAS method and SCF chromatography (SAS-SFC) was proposed to measure solubility for multi-component SCFs by Vorobei et al. [89], which was much faster than the majority of existing methods. Recently, Pokrovskiy et al. [90] demonstrated the applicability of SAS-SFC to the investigation of both selective precipitation from solution and particle size tuning in SAS process using lower dicarboxylic acids as model objects. They found that solubility data were in good agreement with the results of selective crystallization, and difference in solubility gave rise to a selective precipitation using the SAS method. This means that, whenever concentration of an acid is below its solubility in scCO_2_-solvent mixture as predicted by SAS-SFC method, its precipitation does not occur. The same tool also provides the possibility to calculate supesaturation values in SAS process, which can be handy in analyzing concentration dependences of particle size for different initial solvents.

The developed mathematical model can significantly reduce the number of experimental trials required for process design, optimization, and control. A two-dimensional population balance model with solvent removal kinetics was developed by Muthancheri et al. [91] to predict the dynamic behavior of carbamazepine form II crystals produced by a GAS process. The model was able to accurately predict the behavior of crystal size distribution data with a change in process operating conditions. For example, the model was able to accurately predict the increase in crystal size (and size distribution) observed experimentally at a high temperature. A comparison of the model prediction with the experimental results also proved that the model was useful to study the time evolution of aspect ratio, average crystal length, and solute concentration in the solution.

Cardoso et al. [92] provided a mathematical methodology capable of predicting the size and nucleation rate of micro/nano-particles using the Curtet number as a proposal for increasing the scale in laboratory processes. They indicated that Curtet number could be a chosen scaling criterion for validating the results with the same operating conditions, but different volumes of the precipitation chambers. The Curtet number was directly related to the turbulence and indirectly to the particle size. In general, the higher the speed of the jet, the lower the Curtet number, meaning that higher speed might lead to smaller particles.

Clercq et al. [93] brought an innovative molecular modeling approach to the field of SAS crystallization, which helped target the formation of a chosen habit by a modification of the solvent nature or by adding a habit modifier. In their study, attachment energies were calculated in order to model the *in vacuo* crystal habit of different polymorphic forms of sulfathiazole, and then adsorption energies of solvent molecules on each face of these polymorphic forms were calculated with different solvents. In general, using solvents that do not adsorb on the crystal faces, the habit experimentally obtained is similar to that obtained by modeling *in vacuo*; conversely, using a solvent adsorbs on one or several crystal faces, the habit experimentally obtained will be different from the theoretical one.

In the study of Sierra-Pallares et al. [94], a numerical model was developed for analyzing phenomena associated with mixing within the SAS precipitator. In the SAS process, mixing at the microscale is a significant parameter for the design of precipitators to obtain desired particles. In their work, different nozzles were tested and the predictions matched well with experimental data in all the cases studied. This model helped get an insight into mixing dynamics and its influence on the final particle size distribution.

In the study of Cardoso et al. [95], a computational fluid dynamics model coupled to a population balance equation were applied to evaluate the influence of the precipitation chamber volume on the particle size, which proved to be an efficient approach to gaining a better understanding of the production of micro/nanoparticles through the SAS process. This model was able to predict the mean nanoparticle diameter of model drug with an error of 7%. And in the case study, the use of chamber geometry with larger axial length and smaller diameter led to larger particles being precipitated due to the flow pattern promoted by the jet interaction.

## 5. Conclusions and Perspectives

In the pharmaceutical field, SMS are widely prepared by various approaches to mitigate the solubility, stability, and manufacturability of the final drug form. Recently, the SAS process has been proposed greatly for SMS production, due to its capacity in control and modification of the solid-state properties of APIs. Numerous studies have demonstrated that the SAS process can effectively produce desired SMS, especially the pharmaceutical co-crystals and solid dispersions. The solid-state properties of APIs can be well designed by controlling the operating conditions, especially with the help of on-line monitoring and computational techniques, and knowledge about the underlying process mechanisms.

Continuous manufacturing of pharmaceuticals is promising in comparison to traditional batch manufacturing in terms of product quality and manufacturing costs. The developed SAS process shows its potential in the transformation from batch to continuous mode. For instance, the semi-continuous SAS process can fulfill the continuous manufacturing by using multiple precipitation vessels with intelligent pipeline switching. Moreover, the continuous SAS process is compatible with existing spray drying equipment, which removes the need for an increase in capital expenditure in completely new facilities.

Despite the novelty and advantages to produce desired SMS, the SAS process has not been implemented so far at scale in the pharmaceutical industry. Further researches about the scale-up, continuous manufacturing and economic evaluation still need to be conducted. With continuous researching, the SAS process will grow in the pharmaceutical industry. In fact, several companies can now ensure the scale-up study, the production of clinical batches and even the production of commercial batches [17,96], such as CrystecPharma [97], Aphios [98], Sup Eng [99], Lavipharm [100], etc. We believe that SAS process will give a large contribution to the scalable and continuous manufacturing of desired SMS in the near future.

## Figures and Tables

**Table 1:** Examples of pharmaceutical co-crystals prepared via the SAS process.

API/Co-former Method	Operating Parameters ^a^	Solid-State Properties	Ref.
Itraconazole/L-malic acid GAS	*R* = 1:1 mass ratio, *F_CO_*_2_ = 1 g/min *C* = 50 mg/mL, *V* = 10 mL THF *p* = 10.3 MPa, *T* = 40 °C	(a) Contain a certain amorphous material; (b) Slightly larger than those produced from *n*-heptane.	[35]
Naproxen/nicotinamide GAS	*R* = 1:2–3:1 molar ratio *C* = 12–74 mg/mL, *V* = 40 mL acetone *p* = 10 MPa, *T* = 35 °C *F_CO2_* = 2–20 g/min Stirring speed = 60–500 rpm	(a) The same hydrogen-bond network and stoichiometry than co-crystals produced by cooling crystallization, grinding or evaporation techniques; (b) The co-crystal purity was affected by *R*; (c) Pure co-crystals with sizes < 180 μm was obtained.	[46]
Carbamazepine/nicotinamide GAS	*R* = 1:2 or 1:1 molar ratio *V* = 6–8 mL ethanol, *p* = 11 MPa *T* = 40 °C, *F_CO2_* = 90–95 mL/min	(a) The co-crystals were needle shaped; (b) 2.5-fold increase in dissolution rate.	[50]
Mefenamic acid (MEF)/paracetamol GAS process	*R* = 3:1–5:1 mass ratio *C* = 70–90% MEF saturation *V* = 5 mL acetone, *T* = 25 °C−45 °C *p* = 9 MPa, *F_CO2_* = 10 mL/min	Optimized co-crystals improved the MEF dissolution rate by 6.0, 5.3 and 2.3-fold when compared to pure MEF, sieved co-crystals prepared by an evaporation technique and sieved marketed combination drugs, respectively.	[51]
Resveratrol/isoniazid or nicotinamide GAS	*R* = 1:1 molar ratio *V* = 15–25 mL ethanol *p* = 9 MPa, *T* = 45 °C *F_CO2_* = 10 g/min	(a) Their crystal structures similar to those reported; (b) Had non-cocrystallized components; (c) Showed a higher dissolution rate than those obtained by liquid anti-solvent technique.	[52]
Paracetamol/trimethylglycine Semi-continuous	*R* = 1:1 molar ratio, *F_CO2_* = 30 g/min DCM/methanol (1:4–1:1, *v/v*) *C* = 10–40 mg/mL, *p* = 9–12 MPa *T* = 35–50 °C, *F_s_* = 0.5–1.6 mL/min	(a) Mean particle size < 10 μm, much smaller than those obtained using the ball milling process; (b) Enhanced dissolution rate and tableting performance; (c) Residual solvent contents were less than ICH limits.	[31]
Carbamazepine (CBZ)/saccharin Semi-continuous	*R* = 1:1 molar ratio methanol, ethanol, DCM, DMSO *F_s_* = 1 mL/min, *D* = 100 μm *C_CBZ_* = 15–30 mg/mL, *F_CO2_* = 20 g/min *T* = 40.0–60.0 °C, *p* = 10.0–15.0 MPa	(a) Pure co-crystal polymorph I was obtained, but pure co-crystal polymorph II could not be obtained; (b) Showed the same crystal structure and morphology as those previously obtained by other methodologies; (c) Without the presence of homo-crystals and solvent free.	[48]
Naproxen/ nicotinamide Semi-continuous	*R* = 2:1 molar ratio, Acetone *C* = 40 mg/mL, *F_s_* = 2–13 mL/min *T* = 37 °C, *p* = 10 MPa *F_CO2_* = 7–59 g/min	(a) The same hydrogen bond interactions and crystal structure than co-crystals obtained by other techniques; (b) A thin plate-like morphology and a size 20 μm−1 mm; (c) At high *F_CO2_*/*F_s_*, had homo-crystals of naproxen.	[49]
5-Fluorouracil/urea, thiourea or pyrazinamide Semi-continuous	Methanol, *C_5-Fu_* = 2.5–5 mg/mL *F_s_* = 1 mL/min, *F_CO2_* = 20 g/min *p* = 7–15 MPa, *T* = 40 °C, *D* = 100 μm	(a) Pure 5-Fu/urea co-crystals were obtained; (b) 5-Fu/pyrazinamide co-crystal did not be obtained; (c) There were 5-Fu homo-crystal impurities in the precipitate of 5-Fu/thiourea co-crystals.	[53]
Diflunisal/nicotinamide Semi-continuous	*R* = 2:1 molar ratio, ethanol, acetone *C* = 18.64–37.28 mg/mL, *D* = 100 μm *T* = 35- 40 °C, *p* = 10.0–12.0 MPa *F_s_* = 1 mL/min, *F_CO2_* = 20 g/min	(a) Same crystal structure to those obtained by liquid assisted ball mill grinding and solution crystallization; (b) Needles with uniform width and variable length; (c) Improved diflunisal release slightly.	[54]
Indomethacin/saccharin AAS and ASES	Coaxial nozzle (a small mixing chamber~30 μL, *D* = 200 μm) *p* = 6–12 MPa, *T* = 50–70 °C *C* = 4.35 mg/g, *F_s_*/*F_CO2_* = 0.03–0.19 g/g	(a) Pure co-crystals with different morphologies and sizes (0.2–5 μm) were obtained; (b) AAS process: nearly spherical particles; (c) ASES process: larger and elongated particles.	[40]

^a^*R* = the ratio of API/co-former, *V* = solvent volume, *T* = temperature, *p* = pressure, *C* = solute concentration, *F_CO2_* = scCO_2_ dosing rate, *D* = nozzle diameter, *F_s_* = solution flow rate, THF = tetrahydrofuran, DCM = dichloromethane, DMSO = dimethylsulfoxide.

**Table 2:** Examples of solid dispersions prepared via the SAS process.

API/Carrier ^a^ Method	Operating Parameters ^b^	Solid-State Properties	Ref.
Oridonin/ PVP K17 GAS	ethanol *p* = 14 MPa, *T* = 55 °C	(a) Large and adhesive particles, amorphous form; (b) Improved drug dissolution rate greatly; (c) 26.4-fold improvement in the absorption of oridonin.	[61]
Mefenamic acid (MEF)/PVP K30 GAS	acetone/ethanol, *F_CO2_* = 10 mL/min *R* = 1:2–1:0.5 (*w/w*) *C* = 50–75% MEF saturation *p* = 9 MPa, *T* = 25–35 °C	(a) Porous and irregularly shaped; (b) *R*, *C* and *T* affected morphology and size greatly; (c) Possessed the crystalline structure of MEF; (d) Improved the drug dissolution rate greatly.	[65]
Theophylline (THEO)/ PEG 4000 GAS	*V* = 5 mL ethanol/DCM *R* = 2:3–2:7 (*w/w*), *C_THEO_* = 1–2 wt.% *T* = 25–45 °C, *p* = 9 MPa *F_CO2_* = 10 mL/min	(a) Contained some rectangular shape of THEO particles; (b) The excess drugs on the composites can be removed effectively by washing with ethyl acetate; (d) Improved the drug dissolution rate greatly.	[66]
Tadalafil/ PVP K30 Semi-continuous	*R* = 1:4 molar ratio, ethanol/DCM *F_s_* = 1 mL/min, *F_CO2_* = 50 g/min *C* = 5–15 mg/mL, *T* = 40.0–50.0 °C *p* = 9.0–15.0 MPa	(a) Mean particle size 200–900 nm, affected by *T*,*C*, and *p*; (b) C = 15 mg/mL, irregular, micron-sized crystalline particles were obtained, resulting in low dissolution rate; (c) C = 5–10 mg/mL, spherical, nano-sized amorphous particles were obtained, resulting in fast dissolution rate.	[67]
Nimesulide (NIM)/HPMC and PVP K30 Semi-continuous	DCM/methanol (1:1–3:1, *v/v*) *R*(NIM/HPMC/PVP, mass ratio) = 1:1–4:0–2 *C* = 25–55 mg/mL, *F_s_* = 2 mL/min *D* = 100 μm, *F_CO2_* = 100 mL/min *T* = 40 °C, *p* = 8 MPa	(a) Existed in crystal forms or amorphous, *R* and solvent ratio affected the particle solid-state properties greatly; (d) Spherical, micro-sized amorphous particles were obtained, resulting in fast and high dissolution rate; (e) The residual solvents were far below the ICH limits; (f) Amorphous NIM was stable during 12-month storage.	[32]
Itraconazole (ITZ)/HPMC Semi-continuous	methanol/DCM, *D* = 70–650 μm *R* = 3:1–1:4 (*w/w*), *C* = 1–5 mg/mL *T* = 35–55 °C, *p* = 9–17 MPa *F_s_* = 0.5–5 mL/min, *F_CO2_* = 50 g/min	(a) Enhanced ITZ solubility from 4.4 μg/mL to 108.5 μg/mL; (b) Spherical, micro-sized amorphous particles were obtained, resulting in fast and high dissolution rate; (c) Hydrogen bond interaction was formed between HPMC and ITZ, hindering the recrystallization of dissolved ITZ.	[33]
Irbesartan/ Pluronic^®^ F-127 Semi-continuous	Ethanol, *R* = 1:1 (*w/w*) *p* = 10–20 MPa, *T* = 40–70 °C *F_s_* = 0.2–2 mL/min	(a) Spherical, amorphous particles with size 97 ± 19 nm; (b) The dissolution was 13 times more than the pure drug.	[62]
Diclofenac/zein Semi-continuous	DMSO, *R* = 1:5–1:30 (*w/w*) *C* = 30–50 mg/mL, *p* = 9 MPa *T* = 40 °C, *D* = 100 μm *F_s_* = 1 mL/min, *F_CO2_* = 30 g/min	(a) Spherical, amorphous microparticles were obtained with mean diameters 0.416–1.308 μm; (b) The drug release is slower as *R* decreases; (d) When *R* = 1:30, prolonged the drug release successfully.	[63]
Diclofenac or theophylline/Eudragit L100-55 Semi-continuous	DMSO, *R* = 1:10–1:20 (*w/w*) *C* = 20–50 mg/mL, *D* = 100 μm *p* = 9–15 MPa, *T* = 40 °C *F_s_* = 1 mL/min, *F_CO2_* = 30 g/min	(a) *R*, *p* and *C* affected the morphology and size greatly; (b) Spherical, amorphous microparticles were obtained; (c) The drug release delayed up to 28 and 57 times for diclofenac and theophylline, respectively.	[64]
Chrysin (CHS)/PVP Semi-continuous	acetone/ethanol, *R* = 1:4 (*w/w*) C_CHS_ = 1–3 mg/mL, *F_s_* = 1 mL/min *T* = 40–60 °C, *p* = 12 MPa	(a) Spherical, crystalline particles with an average size of 273.7 nm ± 38.9 nm to 958.8 nm ± 83.2 nm were obtained; (b) The dissolution rate was 2.8 times higher than pure CHS;	[68]
Curcumin (CUR)/PVP Semi-continuous	acetone/ethanol, *R* = 2–4 wt% PVP *T* = 40–60 °C, *p* = 8–12 MPa *F_s_* = 0.5 mL/min, *F_CO2_* = 15 mL/min	(a) Spherical particles with size < 1 μm were abtained; (b) The PVP addition enhanced the CUR dissolution in distilled water significantly.	[69]

^a^ PVP = polyvinylpyrrolidone, PEG = polyethylene glycol, HPMC = hydroxypropylmethyl cellulose. ^b^ *R* = the ratio of API/carrier, *V* = solvent volume, *T* = temperature, *p* = pressure, *C* = solute concentration, *F_CO2_* = scCO_2_ dosing rate, *D* = nozzle diameter, *F_s_* = solution flow rate, THF = tetrahydrofuran, DMSO = dimethylsulfoxide, DCM = dichloromethane.

## Data Availability

Not applicable.
